# The Association of Waiting Time with Postoperative Health Care Use and Sickness Absences: A Register Study in Finland

**DOI:** 10.1007/s10926-025-10336-w

**Published:** 2025-10-15

**Authors:** Lauri Vähätalo, Mikko Korhonen, Anna Siukola, Elina Kervinen, Klas Winell, Tiia Reho, Mervi Viljamaa, Riitta Sauni

**Affiliations:** 1 Faculty of Medicine and Health Technology, Tampere University, Tampere, Finland; 2 Conmedic Oy, Espoo, Finland; 3 Pihlajalinna Lääkärikeskukset Oy, Tampere, Finland

**Keywords:** Musculoskeletal disease, Elective surgical procedure, Waiting list, Sick leave, Health services, Occupational health services

## Abstract

**Purpose:**

The aim of this study was to examine the length of waiting time in elective knee, shoulder, hip, and lower back surgery, and how it was associated with sickness absence, use of health care, and work ability support services during 1 year after the surgery.

**Methods:**

The patients (*n* = 429) were grouped by the elective surgery they had undergone. Waiting time was calculated as the time between the day of referral and the day of operation. The register data were analyzed with general linear regression.

**Results:**

The median waiting time for a knee operation (*n* = 287) was 98 days, for a shoulder operation (*n* = 105) 39 days, for a hip operation (*n* = 19), 177 days and for a lower back operation (*n* = 18) 114 days. The use of work ability support services increased after all the operations. A prolonged waiting time (> 90 days) for knee and shoulder operations was associated with the amount of sickness absence (IRR: 1.09, 95% CI 1.06–1.12; IRR: 0.86, 95% CI 0.82–0.89, respectively), secondary health care visits (IRR: 1.81, 95% CI 1.33–2.47; IRR: 2.28, 95% CI 1.50–3.47, respectively), and occupational curative care visits (IRR: 2.06, 95% CI 1.30–3.29) for shoulder operations. No association was found between a prolonged waiting time and work ability service visits during the year after the surgery.

**Conclusion:**

The association of waiting time length with postoperative sickness absences and health care visits calls for taking actions to reduce the waiting times. Waiting should be considered as an active time aiming to prepare for the operation and the recovery.

**Supplementary Information:**

The online version contains supplementary material available at https://doi.org/10.1007/s10926-025-10336-w.

## Introduction

Delays in providing treatment are regarded as a major challenge in European countries [1]. Long waiting times for elective operations may for example decrease the quality of life [2], they lead to poorer health gains from the surgery [3, 4], and they create a financial burden for the patient [5]. Waiting time depends on multiple factors such as health care resources [6], the operation [7], and the service provider’s sector (public or private) [8]. Even a patient’s social status has been shown to affect the length of the waiting time [9].

An international comparison of the reasons for long waiting times is complicated due to differences in national legislation, national treatment guidelines, national health care systems, and capabilities to provide health care [10]. There are also technical and measurement related difficulties affecting the international comparison of waiting times, such as how the waiting time is defined, how waiting time is reported (e.g., average, median, 95 percentile, 90 percentile or other) and what countries measure and publish the waiting times [11]. Similar difficulties persist when a comparison is made on a national level [12].

In Finland secondary health care must assess the patients’ need for care within three weeks after the referral to secondary health care, diagnostic assessment must happen within three months, and surgery, if deemed necessary, must happen within three to six months. Six months is accepted if the operation is non-urgent or if the operation requires the participation of oral health care specialist (13). This results in the maximum care guarantee being nine months (270 days). Despite the increasing number of patients waiting for elective surgery in general, the waiting time for hip and knee replacement surgery has decreased in Finland between 2014 and 2024 due to political guidance [14]. However, during recent years, the number of persons waiting for elective surgery in secondary health care has steadily increased and the effect that COVID-19 outbreak had on the waiting time can still be seen on elective surgery queues in Finland [12, 15].

The type of operation that is being waited for and the waiting time have been shown to affect the patient’s health and self-reported functioning [16]. In a two-year follow-up study among knee arthroplasty patients, the pain level was shown to increase and the quality of life (based on the health-related quality of life -questionnaire) to decrease as the waiting time prolonged [17]. In addition, the health gain from the operation has been shown to decrease slightly among knee and hip replacement patients per every waited week [4].

Despite the vast amount of research underlining the negative effects that a long waiting time has, conflicting findings have also been reported. Tuominen and colleagues [3] showed that knee replacement patients with a shorter waiting time had higher weekly medication costs at admission and reached higher health-related quality of life scores 1 year after the surgery, but 1 year postoperatively, the scores were higher among those who had waited longer. They also showed that the waiting time did not increase the social or health care service use between the time of the decision to treat the patient and the operation day [3]. In regard to the preoperative waiting times, no association was found between the waiting time and an increase in pain levels among osteoarthritis patients waiting for orthopedic consultation or operation [18]. The contradictory results, together with the implications that long waiting time has for the working-age population, call for more research—particularly from the occupational health care (OHC) perspective since OHC has the means to support the working-aged population’s work ability before and after the operation.

The aim of this study was to examine the length of waiting times for certain elective musculoskeletal surgeries and examine whether there is an association between waiting time and sickness absence, the use of secondary health care, occupational curative care, and work ability support services during the year after elective orthopedic surgery.

## Methods

### The Study Setting

#### The Finnish Health Care System and OHC

OHC’s primary function is to promote and support work ability. It is mandatory for the employer to provide preventive OHC [19]. Despite being mandatory, only approximately 90 percent of employees in Finland have access to OHC. From those, approximately 94 percent have access to OHC curative care [20]. The content of curative care may vary greatly between employers and are based on the contracts between the OHC provider and the employer. Having access to curative care does not restrict the patient from using public primary health care or private health care. OHC specialists may also refer patients to public primary care and secondary care if it is seen to be necessary. In Finland employers mostly provide OHC services by purchasing them from private health care providers [20] and rarely have their own OHC specialists, unlike in many European countries [21].

Primary care is provided by three health care sectors in Finland: public, occupational, and private health care. An employee can receive work ability support from OHC if the employee’s work ability has decreased. The patients’ work ability support in OHC often already begins prior to surgery and continues as return-to-work (RTW) support after the surgery. Work ability support may include, for example, workplace job accommodation or finding a new or alternative job role for the employee in the workplace to meet the employee’s individual needs [22–24].

### The Formation of the Study Population

The study population consisted of employees (*n* = 1743) of the client companies of an OHC service provider with a nationwide service network. Hereafter, we refer to the study population as *the patients* or *the OHC patients*. The patients had undergone one of the following elective surgeries in secondary health care between 2019 and 2021 and could be followed from registers between 2018 and 2022. The operations were gathered from the operation coding list provided by the Finnish Institute for Health and Welfare [25] who also provided the operation data. The operations (with procedure codes and the number of patients displayed in parentheses) were the following: (1) posterior fusion of the lumbar spine with fixation (of 2–3 vertebrae) (NAG62) or the release of hip nerve plexus (ACC57) (*n* = 18), (2) arthroscopic suture or reinsertion of the rotator cuff of the shoulder (NBL05) (*n* = 105), (3) plastic repair of the ligament of the knee, the anterior cruciate (ACL), not using prosthetic material (arthroscopic surgery) (NGE35), or primary total prosthetic replacement of the knee joint (TKA) without a patellar part (sliding prosthesis) (NGB20) (*n* = 287), and (4) primary total prosthetic replacement of the hip joint using a hybrid technique (NFB40) (*n* = 19). The study was conducted as a part of a research consortium. These operations were used to enable collaboration across the research consortium.

The lower back operations were grouped due to small frequencies and for this same reason hip and lower back operations were decided not to be included in the regression models. We concluded that the clinical difference between the posterior fusion of the lumbar spine with fixation (of 2–3 vertebrae) and the release of hip nerve plexus does not cause ambiguity to the results, since we report only descriptive statistics from these two operations. In the knee operations ACL and TKA were combined despite the clinical differences in the operations, since the postoperative RTW times have been shown to be relatively similar: 65–107 days in ACL [26–28] and 62–116 days in TKA [29–31].

**Fig. 1:**
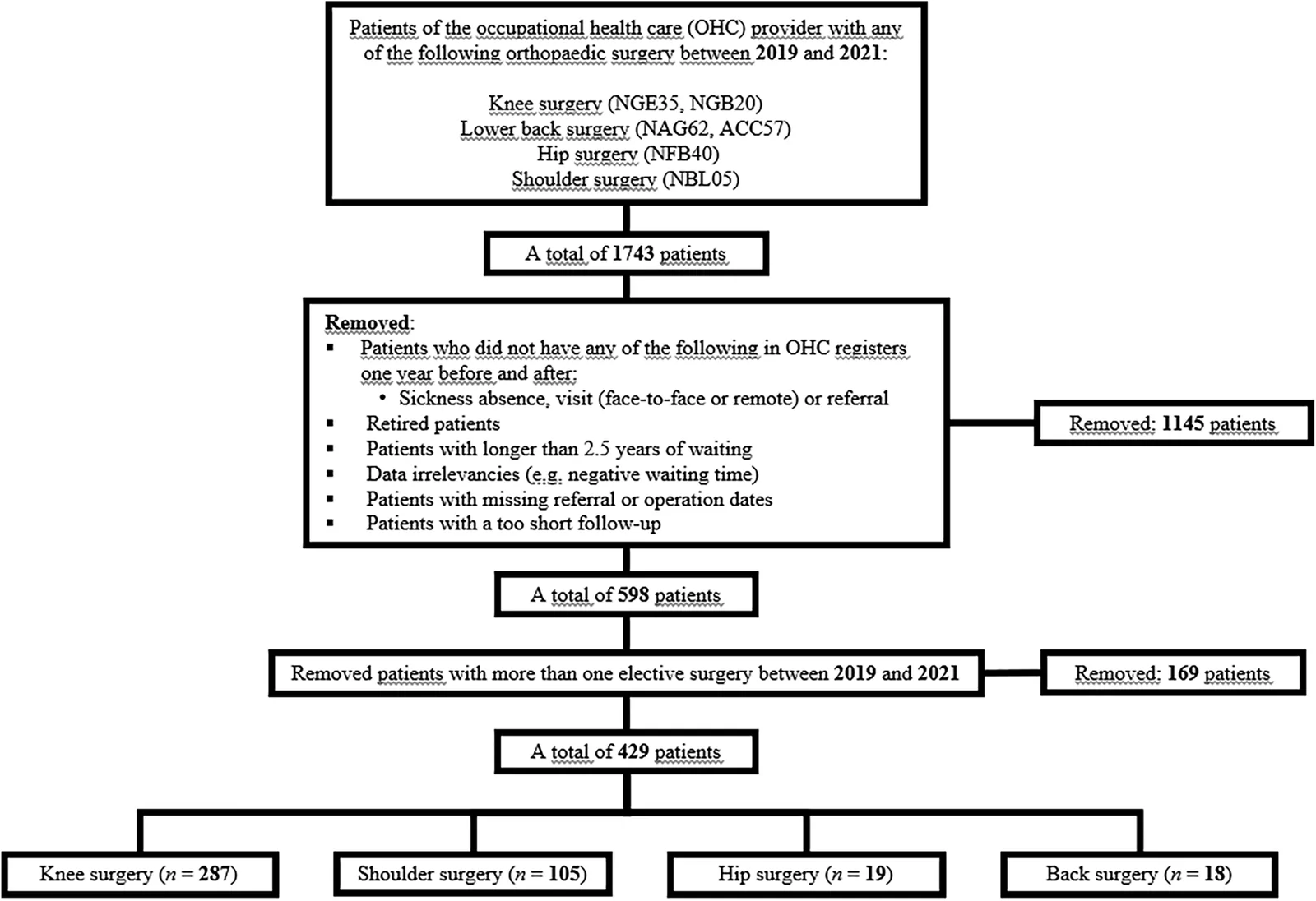
The formation of the study population

The patients also had to have contact (a face-to-face or remote visit), sickness absence or referral information in an OHC provider’s register at least once both 1 year prior to the elective surgery and 1 year after it. In this way, we ensured that the patients had access to the same OHC provider during the study’s follow-up time. We excluded patients with two or more different types of surgery (e.g., knee and hip surgery) to ensure that the associations can be related to a specific operation. Thus, the final population was 429 patients. Figure 1 illustrates the selection and formation of the study population.

### Data Collection

The data contained information from multiple registers: sociodemographic information from Statistics Finland; sickness absence days (> 10 days) from the Social Insurance Institution of Finland (Kela); health care service use, diagnoses, surgical procedures, and elective surgery-related dates (referral date, the date when patient was set to the waiting list, operation date, and discharge day) from the Finnish Institute for Health and Welfare; and work ability support services, visits, diagnoses, and sickness absences (< 10 days) from the OHC provider. The complete data set was combined and pseudonymized by Findata, the Social and Health Data Permit Authority, and Statistics Finland granted secured remote access to the data.

### Waiting Time

The starting point of the waiting time was the date when the referral arrived to secondary health care from OHC or from public or private health care. The waiting ended on the same day that the patient was operated on. Based on the Finnish legislation and the previous literature, we initially divided the waiting time into three categories: a short waiting time; a maximum of 90 days [32, 33], medium waiting time; a maximum of 270 days (Finnish care guarantee) [13] and long waiting time; over 270 days. In the regression models, we decided to use the short waiting time (≤ 90 days) as a reference and combined the medium and long waiting times into a single category: *a prolonged waiting time* (> 90 days) [32]. This was done because of the small number of patients [*n* = 40] with a long waiting time [> 270 days] that would have caused difficulties in the interpretation of the regression models. This division has also been used in the previous research [32].

## Statistical Analysis

The patients were divided into five age categories (18–28, 29–39, 40–49, 50–59, and over 59) and into five professional categories (manual, lower non-manual, upper non-manual, entrepreneurial, and other professions). The patients’ education had four categories (basic education, intermediate [vocational or high school] education, lower tertiary education [a bachelor’s degree], and upper tertiary education [all education above a bachelor’s degree]). The patients’ sociodemographic information was collected from the same year that the patient had the operation.

The used sickness absence diagnoses based on the International Classification of Diseases (ICD-10) [34] were G55.1, M51.1, and M48.0 (for the lower back); S43, S46, and M75 (for the shoulder); M17 and S83.5 (for the knee); and M16 (for the hip). All the diagnosis codes related to sickness absences that the patients had were checked, but only those related to the operation were included in the analysis. Health care service use was followed according to the sector that was visited (OHC curative care or secondary care). Visits in these sectors were combined with the operation by only taking into account those visits with diagnoses that belonged to musculoskeletal diseases (diagnosis codes beginning with M) [34] this way we excluded for this study setting irrelevant sickness absences (e.g., absence due to flu). After initial statistical analyses, we decided not to include public primary health care visits due to the high amount of missing information on visit diagnoses (roughly 40% of the visits were missing this information). After leaving the visits to public primary health care out from the analysis there were no missing data for any variable. All work ability support visits were included. Work ability support visits were handled separately by using the OHC provider’s coding, and work ability support visits did not count as OHC curative care visits.

General linear regression (Poisson family) was carried out to determine whether sickness absence, health care service use, and work ability support after the surgery were associated with the waiting time. Waiting time was an independent variable. Due to the small group size, the hip operation patients (*n* = 19) and lower back operation patients (*n* = 18) were excluded from the regression models.

All the models were adjusted for sex, age, profession, and education. In addition, the models were adjusted for sickness absence 1 year prior to the operation, the health care service visit model was adjusted for health care service visits 1 year prior to the operation, and the work ability support service visit model was adjusted for work ability support service visits 1 year prior to the operation. The reference group varied between the models since some categories had too few patients to be used as a reference group. This is shown in the results table (Supplementary Table 1). The incidence-rate ratio (IRR) with 95% confidence intervals (CIs) was determined. For the regression model, the coefficient was determined by ordinary least squares and the 95% CI was determined by normal distribution approximation. All statistical analyses were conducted with R 4.2.2. *P*-values < 0.05 were considered statistically significant.

## Results

The majority of the OHC patients having elective orthopedic operations were female (Table 1), with the exception of shoulder operations. The patients were mostly over 50 years of age and mainly worked in manual professions or lower non-manual professions for all the operations. The majority of the operated patients had basic or intermediate (vocational or high school) education.

**Table 1:** The sociodemographic information of occupational health care patients having elective musculoskeletal surgery

	Hip operation (*n* = 19)	Shoulder operation (*n* = 105)	Knee operation (*n* = 287)	Lower back operation (*n* = 18)
*Sex*, *n* (%)				
Male	4 (21.1%)	68 (64.8%)	129 (44.9%)	6 (33.3%)
Female	15 (78.9%)	37 (35.2%)	158 (55.1%)	12 (66.7%)
*Age group*, *n* (%)				
18–28	0 (0%)	0 (0%)	9 (3.1%)	0
29–39	0 (0%)	5 (4.8%)	38 (13.2%)	0
40–49	0 (0%)	20 (19.0%)	39 (13.6%)	a
50–59	10 (52.6%)	56 (53.3%)	121 (42.2%)	13 (72.2%)
Over 59	9 (47.4%)	24 (22.9%)	80 (27.9%)	a
*Profession*, *n* (%)				
Manual	a	46 (43.8%)	119 (41.5%)	a
Lower non-manual	10 (52.6%)	38 (36.2%)	102 (35.5%)	6 (33.3%)
Upper non-manual	0	8 (7.6%)	14 (4.9%)	a
Entrepreneur	a	9 (8.6%)	45 (15.7%)	6 (33.3%)
Other	0	4 (3.8%)	7 (2.4%)	0
*Education*, *n* (%)				
Basic	a	15 (14.3%)	22 (7.7%)	a
Intermediate	10 (52.6%)	57 (54.3%)	158 (55.1%)	7 (38.9%)
Lower tertiary	4 (21.1%)	19 (18.1%)	39 (13.6%)	a
Upper tertiary	a	14 (13.3%)	68 (23.7%)	6 (33.3%)

a = the smallest and second smallest frequencies had to be anonymized due to the small number (< 3) of cases

Waiting times varied between the operations (see Table 2). The shortest waiting time was for the shoulder operation where the median waiting time was 39 days, and the longest median waiting time of 177 days was for the hip operations. For all the operations, most of the patients were operated within the Finnish care guarantee time period (270 days).

**Table 2:** The median and average waiting time for elective orthopedic surgery among occupational health care patients in four operations and waiting time groups

	Waiting time				
	Median (days)	Average (days)	≤ 90 days (patients)	91–270 days* (patients)	> 270 days* (patients)
Hip operation	177	220.1	5 (26.3%)	8 (42.1%)	6 (31.6%)
Shoulder operation	39	96.6	71 (67.6%)	27 (25.7%)	7 (6.7%)
Knee operation	98	126.6	136 (47.4%)	125 (43.6)	26 (9.1%)
Lower back operation	114	129.4	a	10 (55.6%)	a

*The Finnish care guarantee time period for elective surgery is 270 days

a = the smallest and second smallest frequencies had to be anonymized due to the small number (< 3) of cases

The patients who had shoulder operations had the most operation-related sickness absence days during 1 year after the operation (Table 3). For all the operations, the average sum of sickness absence days increased during the year after the operation compared with 1 year before the operation. The number of secondary health care visits decreased or stayed the same for all the operations except for lower back operations during the year after the surgery. Visits to OHC curative care decreased or the number of visits stayed the same for all the operations during 1 year after the operation compared with 1 year prior to the operation. For all the operations, work ability support services were used more during the year after the operation than they were during the year prior to the operation. The patients having a lower back operation had the steepest increase in work ability support visits during 1 year after the operation.

**Table 3:** The sickness absence days and the number of health care and work ability service visits during 1 year before the elective orthopedic surgery and during 1 year after it among occupational health care patients

Operation	Median	Average
*Sickness absence (with an operation-specific diagnosis) days* 1 year *before the operation/*1 year *after the operation (in days)*		
Hip operation	0/89	52.2/105.2
Shoulder operation	13/90	34.5/107.4
Knee operation	1/90	27.1/100.8
Lower back operation	0/0	24.0/53.9
*Secondary health care visits (with any M diagnosis) during *1 year* before the operation*/*during *1 year* after the operation*		
Hip operation	2/1	3.4/2.1
Shoulder operation	0/0	1.1/1.1
Knee operation	1/0	1.6/1.0
Lower back operation	2/3	4.3/4.1
*Curative primary OHC visits (with any M diagnosis) during *1 year* before the operation*/*during *1 year* after the operation*		
Hip operation	2/1	2.5/1.2
Shoulder operation	1/0	1.5/0.8
Knee operation	1/1	2.0/1.3
Lower back operation	2/0.5	2.7/0.9
*Work ability support service visits (with any diagnosis) during *1 year* before the operation*/*during *1 year* after the operation (visits per person***)**		
Hip operation	0/3	1.3/3.7
Shoulder operation	0/2	2.0/3.9
Knee operation	1/2	1.8/3.2
Lower back operation	1/5.5	3.4/6.2

The prolonged waiting time (> 90 days) was statistically significantly associated with more sickness absence days during the year after the knee operation (IRR: 1.09, 95% CI 1.06–1.12) (see Table 4). For the shoulder operations, the association was the opposite: the longer the waiting time, the less the patients had sickness absence days during 1 year after the operation (IRR: 0.86, 95% CI 0.82–0.89). A longer waiting time for the shoulder operation was associated with more visits to OHC curative care during the year after the surgery (IRR: 2.06, 95% CI 1.30–3.29). Similarly, a longer waiting time was associated with more visits to the secondary health care after knee operations (IRR: 1.81, 95% CI 1.33–2.47) and after shoulder operations (IRR: 2.28, 95% CI 1.50–3.47) during 1 year after the surgery. We also conducted an analysis without binding the health care visits to the diagnosis codes starting with M. These results showed similar association (data not shown). We did not find any statistically significant association between a longer waiting time and the use of work ability support services.

**Table 4:** The association of waiting time with sickness absence days and the number of visits to secondary, curative occupational health care and work ability support services during 1 year after the operation

	**Knee operation**		**Shoulder operation**	
	IRR (CI 95%)	*p*	IRR (CI 95%)	*p*
*Sickness absence days (with an operation-specific diagnosis) during *1 year* after the operation*				
	**Unadjusted**			
Short waiting time	Ref		Ref	
Prolonged waiting time	1.26 (1.23–1.29)	0.00	0.88 (0.84–0.91)	0.00
	Adjusted*			
Short waiting time	Ref		Ref	
Prolonged waiting time	1.09 (1.06–1.12)	0.00	0.86 (0.82–0.89)	0.00
*Occupational curative care visits (M diagnosis) during *1 year* after the operation*				
	**Unadjusted**			
Short waiting time	Ref		Ref	
Prolonged waiting time	1.19 (0.96–1.46)	0.11	2.14 (1.38–3.33)	0.00
	Adjusted**			
Short waiting time	Ref		Ref	
Prolonged waiting time	1.05 (0.84–1.30)	0.69	2.06 (1.30–3.29)	0.00
*Secondary health care visits (M diagnosis) during *1 year* after the operation*				
	**Unadjusted**			
Short waiting time	Ref		Ref	
Prolonged waiting time	3.07 (2.32–4.07)	0.00	2.05 (1.42–2.97)	0.00
	Adjusted***			
Short waiting time	Ref		Ref	
Prolonged waiting time	1.81 (1.33–2.47)	0.00	2.28 (1.50–3.47)	0.00
*Work ability support service visits (any diagnosis) during *1 year* after the operation*				
	**Unadjusted**			
Short waiting time	Ref		Ref	
Prolonged waiting time	1.11 (0.98–1.27)	0.10	1.31 (1.07–1.60)	0.01
	Adjusted****			
Short waiting time	Ref		Ref	
Prolonged waiting time	0.94 (0.82–1.08)	0.36	1.22 (0.99–1.50)	0.07

*Adjusted for sex, age, education, profession and sickness absences during 1 year prior to the operation

**Adjusted for sex, age, education, profession and occupational curative care visits during 1 year prior to the operation

***Adjusted for sex, age, education, profession and secondary health care visits during 1 year prior to the operation

****Adjusted for sex, age, education, profession and work ability support service visits during 1 year prior to the operation

## Discussion

The patients had more sickness absence days during the year following the operation compared with the year preceding it. The visits to secondary health care decreased after hip and knee operations, stayed approximately the same in lower back operations and increased after shoulder operations. During the year after shoulder, knee, lower back, and hip surgery, the operated patients visited less OHC curative care than during 1 year before the operation. After all the operations, the patients used more work ability support services than during 1 year before the operation.

The changes in all health care related visits are partially self-explanatory. In OHC, the OHC specialist is seen as the musculoskeletal problem begins to cause difficulties in job before the operation. The secondary health care specialist is seen as it has to be ensured that surgery is the most appropriate care for the condition. Finally, the use of work ability support increases as the patients’ needs for rehabilitation and sickness absence are evaluated and RTW is planned after the operation [35]. The increase in sickness absences during the year after the operation is most likely explained by the recovery and rehabilitation periods [36].

The patients had to wait for hip and lower back operations longer than for knee and shoulder operations. Overall, we found that the care guarantee was well implemented for the four operations in this population. However, we could not deduce the effect that the Covid-19 had on this population. In general it is known that the access to elective surgery was disrupted during the pandemic and remained poor for long time after the Covid-19 pandemic [12, 37].

We found statistically significant associations between a longer waiting time and an increased number of visits to secondary health care during 1 year after the knee and shoulder operations. The prolonged waiting time (> 90 days) was also associated with an increase in the number of visits to OHC curative care for the shoulder operations during the year after the operation. These findings may indicate that a longer waiting time cumulates operation-related health issues, and possibly complicates the rehabilitation process [4] and diminishes the benefit that patient experiences from the surgery [38]. We found no statistically significant associations between work ability support service use and a prolonged waiting time nor for the knee or the shoulder operation. This may indicate that the problems related to the patients’ recovery may require more than can be offered by the OHC work ability support services, but this requires more research. To some degree our finding is contradicting with the previous literature where it was found that the waiting time among elderly patients was not associated with an increase in health care service use [39]. However, the OHC patients in this study differ from the elderly patients due to the demands that work sets [40]; thus, a straight comparison with other-than-OHC populations should be done with caution.

The prolonged waiting time (> 90 days) was associated with more sickness absence days after the knee operation, but for the shoulder operations, the association was the opposite. The association between a longer waiting time and sickness absence days after knee operations was anticipated and in line with previous research. A meta-analysis with 89,996 patients showed a significant association between the preoperative waiting time and postoperative joint functioning and quality of life after hip and knee joint replacement surgeries. They also reported that the deteriorating effects most likely occur within the first six months of waiting and may affect clinical outcomes up to 12 months after the surgery [41]. The operation-related medical conditions may cumulate over the waiting time [37] and hinder the recovery process, and prolong the patients’ RTW. A long waiting time also limits the gains from the medical operation [4].

The RTW process after an elective operation has been the target of intensive development in Finland in the past years [35, 42], and there are national clinical practice guidelines for shoulder, knee, hip, and lower back surgery [43–45]. In addition to the waiting time, it has been shown that the timing of the surgery is associated with the surgery’s outcome [41, 46]. Unfortunately, this could not be considered in our study since we did not know when the orthopedic health issue had begun nor how severe the patients’ conditions were.

The association between a longer waiting time and fewer sickness absence days for shoulder operations 1 year after the operation was unexpected and may be due to several reasons. The first is the minimal, clinically important difference, which refers to a minimal difference in terms of the benefits that the patient experiences after a medical intervention [47]. This can be studied, for example, by comparing the pain level before and after the operation [48]. We did not have a pre- or post-operative information regarding the quality of life or pain levels. For this reason, we couldn’t assess the minimal clinical importance. Additionally, the longer waiting time for a shoulder operation could have actually been good for some patients since it has been shown that, for some shoulder conditions, non-operative treatment is as effective, or almost as effective, as operative treatment [49, 50]. Also the Finnish current care guidelines for rotator cuff related issues recommend to primarily apply conservative care before opting for surgery, if the rotator cuff trauma is not acute [43]. The relatively short median waiting time (39 days) and the highest average sum of sickness absence days during the year after the operation in the shoulder operation, also hints that the patients might have had an acute trauma or the decision to operate has been done hastily. Thus, we can’t exclude the possibility that some of the shoulder patients in our data might have received low value care and operation [51]. Unfortunately, we did not have access to the patients’ medical records to confirm or elaborate this more. A straight international comparison of waiting times for a shoulder operation is not possible since no previous research regarding the shoulder operation waiting times was found.

More research is needed on how persons can be prepared for operations (e.g., via physical training for the operation) to enhance the recovery process in advance [43], especially if a person has a long waiting time. Also, the possibility that a patient may have had low value operation should receive more attention in the OHC, since the surgery always causes burden for the individual, employer, and the society. The OHC possibilities to assess the necessity of shoulder operation of the OHC patient could be enhanced by easing the consultation practices between the OHC and secondary health care [52] and increasing the knowledge of the current best practices of shoulder related issues. [43].

The finding that a longer waiting time was not associated with work ability support service use during 1 year after the operation may be explained partially by the prioritization that is done in secondary health care and partially by low value operations. The patients that are prioritized often have more acute or severe situations [53], thus they may receive more gains from the operation, and they may use more work ability support afterward when compared with those who have a less acute or severe condition. The patients with less severe and acute conditions might benefit more from not being operated at all and instead receiving more work ability support since their condition could improve even without the surgery [43]. Our results suggest that patients’ need for sickness absence may vary, even within a specific type of operation, depending on the length of the waiting time. This is supported by the previous literature that has shown contradicting results in regard to postoperative pain levels among patients who have had the same surgical operation [16, 18, 54].

From the beginning of 2020, the care path model has been active in Finland. It has changed the process in some secondary health care clinics so that only short sickness absences (of one to two weeks) are recommended to the patient right after the surgery. After that, the patient is guided to OHC for further assistance, work ability support, rehabilitation, and to receive the most suitable amount of sickness absence [35, 42]. We could not confirm which patients in our study had been operated in a secondary health care unit that follows the above-mentioned model. However, our results support the implementation of this kind of controlled RTW [42] as the patients may have individual sickness absence and health care service needs after the elective surgery. It could also be potentially beneficial to test RTW support and more coordinated care pathways between public primary and secondary health care, since not all working-aged persons have access to OHC [20] and musculoskeletal issues may also limit the employability of unemployed [55].

Our results highlight the need for reducing the waiting times for elective orthopedic operations in secondary health care. We also argue that the waiting time for elective surgery should be considered in OHC since it is associated with sickness absence and increased health care service utilization during 1 year after the surgery. Even though the OHC can’t affect the waiting times, the OHC should regard the waiting time as an active period and encourage and support the patient to prepare for the upcoming operation, for example, by doing physical exercises [56]. This could be rather easy to implement since the OHC professionals often assess the patient’s situation during the waiting time [42]. For these reasons the average waiting time for elective musculoskeletal operations should be a point of interest for the OHC professionals, to help them to plan preoperative interventions and postoperative support to avoid the negative outcomes that may actualize due to prolonged waiting time or operations done too quickly.

### Strengths and Limitations

To our knowledge, this is the first time when the association of waiting time, sickness absence, OHC curative care and secondary health care visits, and work ability support visits before and after orthopedic surgery have been studied from the OHC patients’ perspective in Finland. The data were comprehensive enough to enable the use of regression modeling. The selection of patients was done with caution to avoid misinterpretations and to not include patients that did not have access to OHC. In reporting the results, the STROBE checklist was followed (supplementary Table 2).

The study population only included OHC patients who had specific operations in secondary health care. This limited the sample size. Notably, our data only included employed patients that have relatively good access to health care through OHC and work ability support services [40]. It is also known that work itself is rehabilitating [57], and the workplace community may boost the rehabilitation and create a positive pull back to work [58]. This study population only represents a portion of the Finnish OHC population, which limits the generalization of the results to other contexts. Future studies should include more operations and use bigger populations combining patients from multiple health care sectors to broaden the knowledge on this topic.

The Covid-19 pandemic had a negative impact on the waiting times and care outcomes of patients waiting for elective surgery [37]. Due to the short follow-up and the study setting, we could not deduce the effect that the pandemic might have had on our data.

From the data regarding the referring sector, 90% was missing, therefore, we could not deduce the OHC patients’ care paths before the operation. We assume that there were additional issues related to the quality of life and patient status that we could not recognize from the registers, such as the pain levels. Future studies should include information from medical records, minimum clinical benefit scales, and pain and function scores to provide a more accurate picture of the association of waiting time with sickness absence, and the number of postoperative health care visits. We encourage future studies to use medical records as an additional source of information to allow better understanding on the medical conditions and the care paths that patients had before the operation. This information together with bigger populations should be used to elaborate and validate the findings of our study.

## Conclusion

Waiting time was found to be associated with increased postoperative sickness absences and health care service use in the OHC patients. Waiting times should receive more attention when healthcare services are developed. In OHC the waiting time should be considered as an active phase of preoperative preparation, avoiding passive waiting. By enhancing the consultation practices and information transfer between the OHC and secondary health care the patients with long waiting times could be recognized and they could receive the most appropriate care and support in the OHC.

## Supplementary Information

Below is the link to the electronic supplementary material.

The complete regression models are provided as a supplementary document Supplementary Table 1 (DOCX 45 KB)

The STROBE-checklist is provided as a supplementary document Supplementary Table 2 (DOCX 40 KB)

## Data Availability

Due to legislation and high costs of register combinations in Finland, the data cannot be shared publicly, not even partially. The authors can provide the information indicating how the data set was formed to enable others to apply similar data set from the Social and Health Data Permit Authority.

## References

[1] Eurostat. Unmet health care needs statistics. In: Statistics explained. 2024. https://ec.europa.eu/eurostat/statistics-explained/index.php?title=Unmet_health_care_needs_statistics. Accessed 26 Mar 2025.

[2] Ackerman IN, Bennell KL, Osborne RH. Decline in health-related quality of life reported by more than half of those waiting for joint replacement surgery: a prospective cohort study. BMC Musculoskelet Disord. 2011;23;12:108. 10.1186/1471-2474-12-108. PMC3121657 21605398

[3] Tuominen U, Sintonen H, Hirvonen J, Seitsalo S, Paavolainen P, Lehto M, et al. Is longer waiting time for total knee replacement associated with health outcomes and medication costs? Randomized clinical trial. Value Health. 2010;13;8:998–1004. 10.1111/j.1524-4733.2010.00779.x. 20825622

[4] Nikolova S, Harrison M, Sutton M. The impact of waiting time on health gains from surgery: evidence from a national patient-reported outcome dataset. Health Econ. 2016;258:955–68. 10.1002/hec.3195. 26013773

[5] Fielden JM, Cumming JM, Horne JG, Devane PA, Slack A, Gallagher LM. Waiting for hip arthroplasty: economic costs and health outcomes. J Arthroplasty. 2005; 20;8:990–7. 10.1016/j.arth.2004.12.060. 16376253

[6] McIntyre D, Chow CK. Waiting time as an indicator for health services under strain: a narrative review. Inquiry. 2020. 10.1177/0046958020910305. PMC7235968 32349581

[7] Vohlonen I, Vehviläinen A, Virta L, Husman K, Kröger H, Koistinen V. [Ortopedisiin leikkauksiin liittyvät sairaalahoitojaksot ja sairauspoissaolot työikäisillä vuosina 1998 ja 2013.] Development of length of hospital stay and absence from work among working-age patients undergoing orthopaedic surgery. Suomen lääkärilehti. 2017;72;17:1085–1090a. https://www.laakarilehti.fi/tieteessa/terveydenhuoltoartikkelit/ortopedisiin-leikkauksiin-liittyvat-sairaalahoitojaksot-ja-sairauspoissaolot-tyoikaisilla-vuosina-1998-ja-2013/?public=6c1d45d29a9994b5ce0749390641d957.

[8] González P. On a policy of transferring public patients to private practice. Health Econ. 2005;14;5:513–27. 10.1002/hec.946. 15497171

[9] Carstam L, Rydén I, Gulati S, Rydenhag B, Henriksson R, Salvesen Ø, et al. Socioeconomic factors affect treatment delivery for patients with low grade glioma: a Swedish population-based study. J Neurooncol. 2020;146;2:329–37. 10.1007/s11060-019-03378-7. PMC6971149 31883050

[10] Siciliani L, Moran V, Borowitz M. Measuring and comparing health care waiting times in OECD countries. Health Policy. 2014;118;3:292–303. 10.1016/j.healthpol.2014.08.011. 25217838

[11] Viberg N, Forsberg BC, Borowitz M, Molin R. International comparisons of waiting times in health care-limitations and prospects. Health Policy. 2013;112;1–2:53–61. 10.1016/j.healthpol.2013.06.013. 23895881

[12] Tuominen P. Access to specialized healthcare on 31.12.2022: The care debt for non-urgent specialized healthcare continued to grow in December. In: Statistical report 14/2023. Finnish Institute for Health and Welfare (FIHW/THL). 2023. https://urn.fi/URN:NBN:fi-fe2023041135860. Accessed 26 Mar 2025.

[13] FINLEX. Health Care Act 1326/2010. Ministry of Social Affairs and Health. https://www.finlex.fi/fi/laki/ajantasa/2010/20101326. Accessed 26 Mar 2025.

[14] OECD. Waiting Times for Health Services: Next in Line. In: OECD Health Policy Studies. OECD. 2020. 10.1787/242e3c8c-en. Accessed 26 Mar 2025.

[15] FIHW. Patients waiting for treatment and assessment of the need for treatment by medical specialty. Cube and dashboard interface. https://sampo.thl.fi/pivot/prod/fi/eshjono/jonotilanne/fact_eshjono_jonotilanne. Accessed 10 July 2025.

[16] Hoogeboom TJ, van den Ende CHM, van der Sluis G, Elings J, Dronkers JJ, Aiken AB, et al. The impact of waiting for total joint replacement on pain and functional status: a systematic review. Osteoarthr Cartil. 2009;17;11:1420–7. 10.1016/j.joca.2009.05.008. 19500526

[17] Ho KW, Pong G, Poon WC, Chung KY, Kwok YY, Chiu KH. Progression of health-related quality of life of patients waiting for total knee arthroplasty. J Eval Clin Pract. 2021;27;1:69–74. 10.1111/jep.13388. PMC7891587 32202045

[18] Patten RK, Tacey A, Bourke M, Smith C, Pascoe M, Vogrin S, et al. The impact of waiting time for orthopaedic consultation on pain levels in individuals with osteoarthritis: a systematic review and meta-analysis. Osteoarthr Cartil. 2022;30;12:1561–74. 10.1016/j.joca.2022.07.007. 35961505

[19] FINLEX. Occupational Health Care Act 1383/2001. Ministry of Social Affairs and Health. https://finlex.fi/fi/lainsaadanto/saadoskaannokset/2001/eng/1383. Accessed 26 Mar 2025.

[20] Kela. Kela occupational health care statistics 2020. In: Social Security 2022. Official Statistics of Finland (Kela). 2022. http://urn.fi/URN:NBN:fi-fe2024062758898. Accessed 26 Mar 2025.

[21] Hassard J, Jain A, Leka S. International comparison of occupational health systems and provisions: a comparative case study review. In: DWP research report. Department for Work and Pensions. 2021. https://www.gov.uk/government/publications/international-comparison-of-occupational-health-systems-and-provisions. Accessed 26 Mar 2025.

[22] Kausto J, Mattila-Holappa P, Kaila-Kangas L, Oksanen T, Kivimäki M, Ervasti J. Support measures for work ability and participation in work: self-reporting practices for sick leave, the model of substitute work, work ability coordinator activities, and partial disability benefits in the municipal sector. In: Tietoa työstä. Finnish Institute of Occupational Health. 2020. https://urn.fi/URN:ISBN:9789522619051. Accessed 26 Mar 2025.

[23] Kausto J, Oksanen T, Koskinen A, Pentti J, Mattila-Holappa P, Kaila-Kangas L, et al. ”Return to Work” coordinator model and work participation of employees: a natural intervention study in Finland. J Occup Rehabil. 2021;31:831–9. 10.1007/s10926-021-09970-x. PMC8558281 33829365

[24] Ervasti J, Kausto J, Leino-Arjas P, Turunen J, Varje P, Väänänen A. Effectiveness of interventions to support work ability and prevent work disability: review on effects and cost effectiveness. In: Publications of the government´s analysis, assessment and research activities 2022:7. 2022. http://urn.fi/URN:ISBN:978-952-383-131-5. Accessed 26 Mar 2025.

[25] Lehtonen J, Lehtovirta J, Mäkelä-Bengs P. FIHW-Procedure classification. In: Guidance 4/2013. Finnish Institute for Health and Welfare (FIHW/THL). 2013. https://urn.fi/URN:ISBN:978-952-245-858-2. Accessed 26 Mar 2025.

[26] Arimaa A, Knifsund J, Keskinen H, Kivimäki M, Aalto V, Oksanen T, et al. Return to work following anterior cruciate ligament reconstruction. Acta Orthop. 2022;14;93:554–9. 10.2340/17453674.2022.3139. PMC9205185 35700049

[27] Tiftikci U, Serbest S, Kilinc CY, Karabicak GÖ, Vergili Ö. Return to work in miners following anterior cruciate ligament reconstruction. Pan Afr Med J. 2015;22:173. 10.11604/pamj.2015.22.173.7979. PMC4750887 26918069

[28] Özbek EA, DursunSavran M, Baltacı Ç, Herman ZJ, Zsidai B, Wailing J, et al. Return to work after anterior cruciate ligament reconstruction: a systematic review. Orthop J Sports Med. 2024;12;5. 10.1177/23259671241249086. PMC11092533 38745916

[29] Van Leemput D, Neirynck J, Berger P, Vandenneucker H. Return to work after primary total knee arthroplasty under the age of 65 years: a systematic review. J Knee Surg. 2022;35;11:1249–59. 10.1055/s-0040-1722626. 33472262

[30] Styron JF, Barsoum WK, Smyth KA, Singer ME. Preoperative predictors of returning to work following primary total knee arthroplasty. J Bone Joint Surg. 2011;93;1:2. 10.2106/jbjs.i.01317. 21209263

[31] Tan FBN, Tan CMP, Wong TAKW, Zhang W, Kunnasegaran R. Return to work following knee arthroplasty: a retrospective review in urban Asian population. Eur J Orthop Surg Traumatol. 2024;34;1:397–403. 10.1007/s00590-023-03662-3. 37552287

[32] Tuominen U, Sintonen H, Hirvonen J, Seitsalo S, Paavolainen P, Lehto M, et al. The effect of waiting time on health and quality of life outcomes and costs of medication in hip replacement patients: a randomized clinical trial. Osteoarthritis Cartilage. 2009;17;9:1144–50. 10.1016/j.joca.2009.03.014. 19374960

[33] Hirvonen J, Blom M, Tuominen U, Seitsalo S, Lehto M, Paavolainen P, et al. Is longer waiting time associated with health and social services utilization before treatment? A randomized study. J Health Serv Res Policy. 2007;12;4:209–14. 10.1258/135581907782101606. 17925072

[34] FIHW. The 3rd Finnish Revised Version of the World Health Organization’s International Classification of Diseases (ICD-10). Finnish Institute for Health and Welfare (FIHW/THL). 2011. https://urn.fi/URN:NBN:fi-fe201205085423. Accessed 11 Sep 2025.

[35] Lavikainen P, Heiskanen J, Jalkanen K, Lehtimäki AV, Vehkala S, Kangas P, et al. Effectiveness of the coordinated return to work model after orthopaedic surgery for lumbar discectomy and hip and knee arthroplasty: a register-based study. Occup Environ Med. 2024;8;13:150–157. 10.1136/oemed-2023-109276. PMC10958326 38331568

[36] Brox JI, Skare Ø, Mowinckel P, Brox JS, Reikerås O, Schrøder CP. Sick leave and return to work after surgery for type II SLAP lesions of the shoulder: a secondary analysis of a randomised sham-controlled study. BMJ Open. 2020;10;4:e035259. 10.1136/bmjopen-2019-035259. PMC7170628 32241790

[37] Clement ND, Scott CEH, Murray JRD, Howie CR, Deehan DJ, IMPACT-Restart Collaboration. The number of patients ”worse than death” while waiting for a hip or knee arthroplasty has nearly doubled during the COVID-19 pandemic. Bone Joint J. 2021;103-B;4:672–80. 10.1302/0301-620x.103b.bjj-2021-0104.r1. 33752468

[38] Guo MY, Crump RT, Karimuddin AA, Liu G, Bair MJ, Sutherland JM. Prioritization and surgical wait lists: a cross-sectional survey of patient’s health-related quality of life. Health Policy. 2022;126;2:99–105. 10.1016/j.healthpol.2021.12.006. 34991899

[39] Quan H, Lafreniere R, Johnson D. Health service costs for patients on the waiting list. Can J Surg. 2002;45:34–42. PMC3692702 11837919

[40] Rinne H, Laaksonen M, Blomgren J. Use of outpatient and inpatient health care services by occupation—a register study of employees in Oulu. Finland BMC Health Serv Res. 2022;22;597:1–13. 10.1186/s12913-022-07970-y. PMC9066753 35505398

[41] Cooper GM, Bayram JM, Clement ND. The functional and psychological impact of delayed hip and knee arthroplasty: a systematic review and meta-analysis of 89,996 patients. Sci Rep. 2024;14;1:8032. 10.1038/s41598-024-58050-6. PMC10997604 38580681

[42] Kangas P, Soini S, Pamilo K, Kervinen V, Kinnunen ML. Return to work following hip or knee arthroplasty: a one-year prospective cohort study in participants with direct referral from hospital to occupational health care services. J Occup Rehabil. 2024;35;3:505–19. 10.1007/s10926-024-10218-7. PMC12361289 38896401

[43] Current Care Guidelines. Shoulder tendon issues. In: Current Care Guidelines. The Finnish Medical Society Duodecim. 2022. https://www.terveysportti.fi/apps/dna/ltk/article/hoi50099/search/rotator%20cuff. Accessed 26 Mar 2025.

[44] Current Care Guidelines. Knee and hip osteoarthritis. In: Current Care Guidelines. The Finnish Medical Society Duodecim. 2018. https://www.terveysportti.fi/apps/dna/ltk/article/hoi50054?toc=1133835_bc. Accessed 26 Mar 2025.

[45] Choices in Health Care. Treatment of chronic lower back symptoms with fusion surgery and postoperative rehabilitation (recommendation). In: Choices in Health Care. Finnish Government. 2021. https://palveluvalikoima.fi/kroonisen-lanneselan-oireiston-hoito-luudutus-kirurgialla. Accessed 26 Mar 2025.

[46] Ghomrawi HMK, Mushlin AI, Kang R, Banerjee S, Singh JA, Sharma L, et al. Examining timeliness of total knee replacement among patients with knee osteoarthritis in the U.S.: results from the OAI and MOST longitudinal cohorts. J Bone Joint Surg Am. 2020;102;6:468–76. 10.2106/jbjs.19.00432. PMC7508265 31934894

[47] Cook CE. Clinimetrics Corner: The Minimal Clinically Important Change Score (MCID): a necessary pretense. J Man Manip Ther. 2008;16;4:E82–83. 10.1179/jmt.2008.16.4.82e. PMC2716157 19771185

[48] Draak THP, de Greef BTA, Faber CG, Merkies ISJ, PeriNomS study group. The minimum clinically important difference: which direction to take. Eur J Neurol. 2019;26;6:850–5. 10.1111/ene.13941 PMC6593833 30793428

[49] Goldberg BA, Nowinski RJ, Matsen FA. Outcome of nonoperative management of full-thickness rotator cuff tears. Clin Orthop Relat Res. 2001;382:99–107. 10.1097/00003086-200101000-00015. 11154011

[50] Cederqvist S, Flinkkilä T, Sormaala M, Ylinen J, Kautiainen H, Irmola T, et al. Non-surgical and surgical treatments for rotator cuff disease: a pragmatic randomised clinical trial with 2-year follow-up after initial rehabilitation. Ann Rheum Dis. 2021;80;6:796–802. 10.1136/annrheumdis-2020-219099. PMC8142425 33272959

[51] Raudasoja A, De-implementation of low-value care. Doctoral dissertation. University of Helsinki. 2025. http://urn.fi/URN:ISBN:978-952-84-1174-1. Accessed 19 Sep 2025.

[52] Sarmala S, Vähätalo L, Siukola A, Reho T, Sauni R. [Erikoissairaanhoidon ja työterveyshuollon yhteinen potilas – tiedonsiirto olennaisena osana saumatonta yhteistyötä.] A shared patient between specialized healthcare and occupational health – information transfer as an essential part of seamless collaboration. Lääketieteellinen Aikakauskirja Duodecim. 2024;140:1133–8. https://www.duodecimlehti.fi/duo18323.

[53] Gutacker N, Siciliani L, Cookson R. Waiting time prioritisation: evidence from England. Soc Sci Med. 2016;159:140–51. 10.1016/j.socscimed.2016.05.007. 27183130

[54] Desmeules F, Dionne CE, Belzile E, Bourbonnais R, Frémont P. Waiting for total knee replacement surgery: factors associated with pain, stiffness, function and quality of life. BMC Musculoskelet Disord. 2009;10:52. 10.1186/1471-2474-10-52. PMC2694153 19457252

[55] Vermeulen SJ, Heymans MW, Anema JR, Schellart AJ, van Mechelen W, van der Beek AJ. Economic evaluation of a participatory return-to-work intervention for temporary agency and unemployed workers sick-listed due to musculoskeletal disorders. Scand J Work Environ Health. 2013;39;1:46–56. 10.5271/sjweh.3314 22854773

[56] Blom AW, Artz N, Beswick AD, Burston A, Dieppe P, Elvers KT, et.al. Improving patients’ experience and outcome of total joint replacement: the RESTORE programme. Programme Grants Appl Res. 2016;4(12). 10.3310/pgfar04120. Accessed 26 Mar 2025. 27559567

[57] Modini M, Joyce S, Mykletun A, Christensen H, Bryant RA, Mitchell PB, et al. The mental health benefits of employment: results of a systematic meta-review. Australas Psychiatry. 2016;24;4:331–6. 10.1177/1039856215618523. 26773063

[58] Etuknwa A, Daniels K, Eib C. Sustainable return to work: a systematic review focusing on personal and social factors. J Occup Rehabil. 2019;29;4:679–700. 10.1007/s10926-019-09832-7. PMC6838034 30767151

