# Supplementary material for: The Association of Waiting Time with Postoperative Health Care Use and Sickness Absences: A Register Study in Finland
Source: J Occup Rehabil. 2025 Oct 15;36(4):1099–109. doi: 10.1007/s10926-025-10336-w (PMC13759626; doi:10.1007/s10926-025-10336-w)
Supplement: Supplementary file 1 [file 10926_2025_10336_MOESM1_ESM.docx]

|  | **Sickness absence days (with an operation-specific diagnosis) during one year after the operation** | | | |
| --- | --- | --- | --- | --- |
|  | **Knee operation** | | **Shoulder operation** | |
|  | **IRR (CI: 95%)** | ***p*** | **IRR (CI: 95%)** | ***p*** |
|  | **Unadjusted** | | | |
| Short waiting time | Ref. |  | Ref. |  |
| Prolonged waiting time | 1.26 (1.23–1.29) | 0.00 | 0.88 (0.84–0.91) | 0.00 |
|  | **Adjusted** | | | |
| Short waiting time | Ref. |  | Ref. |  |
| Prolonged waiting time | 1.09 (1.06–1.12) | 0.00 | 0.86 (0.82–0.89) | 0.00 |
| Sickness absences before the operation | 1.00 (1.00–1.00) | 0.00 | 1.00 (1.00–1.00) | 0.00 |
| Male | Ref-. |  | Ref. |  |
| Female | 1.19 (1.15–1.22) | 0.00 | 1.29 (1.23–1.35) | 0.00 |
| Age: 18–28 | Ref. |  | n/a |  |
| Age: 29–39 | 1.44 (1.29–1.61) | 0.00 | Ref. |  |
| Age: 40–49 | 2.29 (2.05–2.56) | 0.00 | 0.67 (0.60–0.74) | 0.00 |
| Age: 50–59 | 2.75 (2.47–3.06) | 0.00 | 0.77 (0.70–0.84) | 0.00 |
| Age: Over 59 | 2.86 (2.57–3.18) | 0.00 | 0.79 (0.72–0.87) | 0.00 |
| Education: basic | Ref. |  | Ref. |  |
| Education: secondary | 0.90 (0.86–0.94) | 0.00 | 1.13 (1.07–1.19) | 0.00 |
| Education: lower tertiary | 0.88 (0.83–0.93) | 0.00 | 0.68 (0.63–0.74) | 0.00 |
| Education: upper tertiary | 0.84 (0.79–0.89) | 0.00 | 0.67 (0.61–0.74) | 0.00 |
| Manual professions | Ref. |  | Ref. |  |
| Lower non-manual professions | 0.86 (0.83–0.88) | 0.00 | 0.97 (0.92–1.03) | 0.31 |
| Upper non-manual professions | 0.59 (0.56–0.62) | 0.00 | 0.68 (0.60–0.77) | 0.00 |
| Entrepreneurial professions | 0.60 (0.55–0.66) | 0.00 | 0.68 (0.60–0.77) | 0.00 |
| Other professions | 1.17 (1.10–1.23) | 0.00 | 1.04 (0.97–1.12) | 0.25 |
|  | **Occupational curative care visits (M diagnosis) during one year after the operation** | | | |
|  | **Knee** | | **Shoulder** | |
|  | **Unadjusted** | | | |
| Short waiting time | Ref. |  | Ref. |  |
| Prolonged waiting time | 1.19 (0.96-1.46) | 0.11 | 2.14 (1.38-3.33) | 0.00 |
|  | **Adjusted** | | | |
| Short waiting time | Ref. |  | Ref. |  |
| Prolonged waiting time | 1.05 (0.84–1.30) | 0.69 | 2.06 (1.30–3.29) | 0.00 |
| Occupational curative care visits one year before the operation | 1.12 (1.08–1.16) | 0.00 | 1.05 (0.96–1.14) | 0.32 |
| Male | Ref. |  | Ref. |  |
| Female | 1.47 (1.14–1.89) | 0.01 | 1.72 (1.00–2.96) | 0.05 |
| Age: 18–28 | Ref. |  | n/a |  |
| Age: 29–39 | 0.38 (0.16–0.91) | 0.03 | Ref. |  |
| Age: 40–49 | 1.32 (0.61–2.83) | 0.48 | 2.68 (0.33–21.66) | 0.36 |
| Age: 50–59 | 1.11 (0.53–2.32) | 0.79 | 3.27 (0.44–24.62) | 0.25 |
| Age: Over 59 | 1.18 (0.56–2.47) | 0.66 | 3.51 (0.46–26.94) | 0.23 |
| Education: basic | Ref. |  | Ref. |  |
| Education: secondary | 0.83 (0.58–1.21) | 0.33 | 0.89 (0.46–1.74) | 0.74 |
| Education: lower tertiary | 0.48 (0.29–0.78) | 0.00 | 1.77 (0.80–3.91) | 0.16 |
| Education: upper tertiary | 0.56 (0.34–0.93) | 0.02 | 0.96 (0.31–2.90) | 0.94 |
| Manual professions | Ref. |  | Ref. |  |
| Lower non-manual professions | 0.92 (0.70–1.22) | 0.57 | 0.65 (0.34–1.24) | 0.19 |
| Upper non-manual professions | 0.72 (0.45–1.14) | 0.16 | 0.56 (0.18–1.75) | 0.32 |
| Entrepreneurial professions | 0.97 (0.45–2.11) | 0.94 | 0.33 (0.04–2.47) | 0.28 |
| Other professions | 0.56 (0.28–1.10) | 0.9 | 1.84 (0.81–4.18) | 0.15 |
|  | **Secondary health care visits (M diagnosis) during one year after the operation** | | | |
|  | **Knee** | | **Shoulder** | |
|  | **Unadjusted** | | | |
| Short waiting time | Ref. |  | Ref. |  |
| Prolonged waiting time | 3.07 (2.32–4.07) | 0.00 | 2.05 (1.42–2.97) | 0.00 |
|  | **Adjusted** | | | |
| Short waiting time | Ref. |  | Ref. |  |
| Prolonged waiting time | 1.81 (1.33–2.47) | 0.00 | 2.28 (1.50–3.47) | 0.00 |
| Secondary health care visits one year before the operation | 1.26 (1.21–1.31) | 0.00 | 1.46 (1.34–1.59) | 0.00 |
| Man | Ref. |  | Ref. |  |
| Woman | 0.96 (0.72–1.28) | 0.79 | 0.94 (0.56–1.59) | 0.83 |
| Age: 18–28 | n/a |  | n/a |  |
| Age: 29–39 | Ref. |  | Ref. |  |
| Age: 40–49 | 3.36 (1.54–7.32) | 0.00 | 1.90 (0.23–15.89) | 0.55 |
| Age: 50–59 | 3.48 (1.67–7.27) | 0.00 | 5.38 (0.72–40.28) | 0.10 |
| Age: Over 59 | 2.44 (1.14–5.24) | 0.02 | 3.82 (0.50–29.04) | 0.20 |
| Education: basic | Ref. |  | Ref. |  |
| Education: secondary | 0.91 (0.58–1.42) | 0.67 | 0.65 (0.38–1.10) | 0.11 |
| Education: lower tertiary | 0.83 (0.47–1.47) | 0.53 | 0.65 (0.33–1.29) | 0.22 |
| Education: upper tertiary | 1.13 (0.63–2.05) | 0.68 | 0.77 (0.28–2.16) | 0.62 |
| Manual professions | Ref. |  | Ref. |  |
| Lower non-manual professions | 0.95 (0.66–1.35) | 0.77 | 0.51 (0.28–0.93) | 0.03 |
| Upper non-manual professions | 0.87 (0.52–1.45) | 0.59 | 1.11 (0.45–2.73) | 0.82 |
| Entrepreneurial professions | 2.31 (1.21–4.40) | 0.01 | 1.25 (0.37–4.23) | 0.72 |
| Other professions | 0.68 (0.27–1.67) | 0.40 | 1.70 (0.86–3.36) | 0.13 |
|  | **Work ability support service visits (any diagnosis) during one year after the operation** | | | |
|  | **Knee** | | **Shoulder** | |
|  | **Unadjusted** | | | |
| Short waiting time | Ref. |  | Ref. |  |
| Prolonged waiting time | 1.11 (0.98–1.27) | 0.10 | 1.31 (1.07–1.60) | 0.01 |
|  | **Adjusted** | | | |
| Short waiting time | Ref. |  | Ref. |  |
| Prolonged waiting time | 0.94 (0.82–1.08) | 0.36 | 1.22 (0.99–1.50) | 0.07 |
| Work ability service visits one year before the operation | 1.07 (1.06–1.09) | 0.00 | 1.08 (1.05–1.11) | 0.00 |
| Man | Ref. |  | Ref. |  |
| Woman | 1.40 (1.19–1.64) | 0.00 | 1.37 (1.06–1.77) | 0.02 |
| Age: 18–28 | Ref. |  | n/a |  |
| Age: 29–39 | 0.94 (0.49–1.83) | 0.86 | Ref. |  |
| Age: 40–49 | 2.84 (1.53–5.27) | 0.00 | 1.51 (0.68–3.33) | 0.31 |
| Age: 50–59 | 2.51 (1.37–4.62) | 0.00 | 2.22 (1.04–4.74) | 0.04 |
| Age: Over 59 | 2.56 (1.39–4.71) | 0.00 | 2.12 (0.97–4.60) | 0.06 |
| Education: basic | Ref. |  | Ref. |  |
| Education: secondary | 1.17 (0.89–1.53) | 0.26 | 1.56 (1.17–2.08) | 0.00 |
| Education: lower tertiary | 1.15 (0.83–1.58) | 0.40 | 0.83 (0.55–1.25) | 0.38 |
| Education: upper tertiary | 0.91 (0.65–1.26) | 0.57 | 0.77 (0.43–1.39) | 0.39 |
| Manual professions | Ref. |  | Ref. |  |
| Lower non-manual professions | 0.79 (0.66–0.95) | 0.01 | 0.60 (0.46–0.80) | 0.00 |
| Upper non-manual professions | 0.57 (0.43–0.77) | 0.00 | 0.15 (0.05–0.44) | 0.00 |
| Entrepreneurial professions | 0.63 (0.37–1.08) | 0.09 | 0.06 (0.01–0.40) | 0.00 |
| Other professions | 0.90 (0.65–1.25) | 0.53 | 0.47 (0.31–0.73) | 0.00 |
